# A 10-Year Study on Larynx Preservation Compared With Surgical Resection in Patients With Locally Advanced Laryngeal and Hypopharyngeal Cancers

**DOI:** 10.3389/fonc.2020.535893

**Published:** 2020-10-15

**Authors:** Xuan Su, Hui-Chan He, Zu-Lu Ye, Da-Lei Zhou, Qing Liu, Xin-Hua Yang, Ya-Kang Long, Tao Tang, Jiang-Jun Ma, Bo-Heng Xu, Wei-Chao Chen, Cai-Yun He, An-Kui Yang

**Affiliations:** ^1^Department of Head and Neck, Sun Yat-sen University Cancer Center, State Key Laboratory of Oncology in South China, Collaborative Innovation Center for Cancer Medicine, Guangzhou, China; ^2^Department of Blood Transfusion, Sun Yat-sen University Cancer Center, State Key Laboratory of Oncology in South China, Collaborative Innovation Center for Cancer Medicine, Guangzhou, China; ^3^Department of Molecular Diagnostics, Sun Yat-Sen University Cancer Center, State Key Laboratory of Oncology in South China, Collaborative Innovation Center for Cancer Medicine, Guangzhou, China

**Keywords:** larynx preservation, laryngectomy, laryngeal cancer, hypopharynx cancer, overall survival, progression free survival

## Abstract

**Background:**

Few reports from China provide confirmed evidence of the effectiveness of the larynx preservation strategy compared with surgery on the treatment of laryngeal and hypopharyngeal cancers. This study assessed the clinical outcomes of patients with locally advanced laryngeal and hypopharyngeal cancers treated with larynx preservation and determined the optimal larynx preservation procedure.

**Methods:**

Data of 1,494 patients treated with total laryngectomy or larynx preservation between 2006 and 2014 were retrieved from the database of Sun-Yat Sen University Cancer Center in Guangzhou, China, and 366 eligible patients were selected for final analysis. The clinical outcomes of 228 patients received total laryngectomy and 138 patients received larynx preservation treatments, which comprises induction followed by radiotherapy and concurrent radio-chemotherapy, were compared.

**Results:**

There was no statistical difference in the 3-, 5-, and 10-year PFS and OS in patients received larynx preservation compared with patients treated with laryngectomy. With respect to T stage, a better overall OS in T2-stage disease (P = 0.036) but poorer PFS (P = 0.005) in T3-stage disease was observed in the larynx preservation group compared with the surgery group in Univariate analysis. T3-stage disease had poorer PFS in multivariable analysis (P = 0.022). With larynx preservation intent, induction chemotherapy followed by radiotherapy showed no advantage in the control of disease progression and survival compared with concurrent chemoradiotherapy. The patient subpopulations who received efficacy assessment after induction chemotherapy exhibited significantly longer PFS and OS compared with those without efficacy assessment.

**Conclusions:**

This is the largest sample size study on larynx preservation treatment for laryngeal and hypopharyngeal cancers in China. Our results indicated that larynx preservation treatments did not jeopardize the survival of patients with advanced resectable laryngeal or hypopharyngeal cancers. Efficacy assessment should be emphasized in induction chemotherapy.

## Introduction

Laryngeal and hypopharyngeal cancers are often analyzed in combination because of their adjacent anatomical location, similarity in treatment strategies, and effects on the patients’ quality of life. These cancers frequently occur in elderly people. Most patients present with signiﬁcant comorbidities and advanced-stage disease. Laryngeal and hypopharyngeal cancers are traditionally treated with surgery, most often total laryngectomy, followed by post-operative radiotherapy (1). Nevertheless, this strategy inevitably destroys the function of speech and swallowing, having a negative impact on patients’ quality of life.

Since the early 1990s, the larynx preservation approach has been developed to avoid total laryngectomy (2). Several critical randomized clinical trials, including the VA study (2), EORTC 24891 trial (3, 4), and RTOG 91-11 trial (5, 6) proved the advantage of chemotherapy combined with radiotherapy in preserving larynx. Encouraging results of these trials led to a change in the treatment guidelines for locally advanced laryngeal and hypopharyngeal cancers (7, 8).

In China, the larynx preservation strategy began to be applied for laryngeal and hypopharyngeal cancers in the early 2000s. Various therapeutic options have been administered, including radiotherapy (RT) alone, concomitant radio-chemotherapy (RT/CT), induction chemotherapy (ICT) followed by RT (ICT-RT), or RT/CT (ICT-RT/CT), and the combination of RT with anti-epidermal growth factor receptor (anti-EGFR) therapy. However, few reports from China provide confirmed evidence of the effectiveness of the larynx preservation strategy compared with surgery (9). Therefore, an overview of the short- and long-term outcomes of larynx preservation treatment from China is essential. It is also urgent to clarify which option for larynx preservation exerts the most positive effect in China.

Sun Yat-Sen University Cancer Center (SYSUCC) is the largest integrated center in southern China for cancer-related care, where robust researches on cancers are carried out. The goal of this study is to perform a systematic review of current approaches in laryngeal and hypopharyngeal cancers treatment in SYSUCC. We scanned the data for all patients with resectable locally advanced laryngeal and hypopharyngeal cancers in SYSUCC since the first patient was treated by larynx preservation strategy on August 21^st^, 2006. Strict inclusion and exclusion criteria were followed. Routine follow up was performed using the Clinical Follow-up Department of SYSUCC every six months, and a final follow up for confirmation was conducted by two researchers (X. Su and C.Y. He). We compared the clinical outcomes of patients treated with curative intent either with or without larynx preservation approaches, and explored the significance of induction chemotherapy in larynx preservation strategy.

## Materials and Methods

### Screening of Patients With Resectable and Locally Advanced Laryngeal and Hypopharyngeal Cancers

Clinical records of all patients who presented with laryngeal and hypopharyngeal cancers from 21 August 2006 to 24 September 2014 in SYSUCC, Guangzhou, China were retrospectively reviewed. The inclusion criteria were as follows: 1) pathologically diagnosed laryngeal or hypopharyngeal squamous cell carcinoma, newly treated in the cancer center; 2) stage III–IVA locally advanced but resectable laryngeal or hypopharyngeal cancer (according to the American Joint Commission on Cancer 7th edition), without synchronous tumors or distant metastases; and 3) patients received non-surgical preservation approaches or underwent total laryngectomy with complete clinical and pathological records. The exclusion criteria were as follows: 1) stages I and II or unresectable stage IV disease; 2) presence of second primary tumors; 3) presence of distant metastases at their first visit at SYSUCC; 4) without complete clinical and pathological records; 5) patients received conservation partial laryngectomy; and 6) patients did not complete a full course of larynx preservation approach because of complications or because they refused or abandoned the treatment. The protocols were approved by the Ethics Committee of the Sun Yat-Sen University Cancer Center, Guangdong, China. Written informed consent was obtained from all patients at their first visit. The doctor introduced the treatment plan to the patients, including surgery, mainly total laryngectomy, or larynx preservation treatment. The patients and their families finally chose the treatment voluntarily.

### Induction Chemotherapy and Evaluation of Tumor Response

The regimens consisting of PF (platinum plus fluorouracil), TP (docetaxel plus platinum), and TPF (docetaxel, platinum and fluorouracil). The doses of corresponding regimens were as followings: docetaxel 60 mg/m^2^, cisplatin 60 mg/m^2^, and 5-fluorouracil 600 mg/m^2^. After two treatment cycles, clinical tumor response was assessed only by radiological evaluation, including computed tomography or magnetic resonance imaging scan of the neck. Only those patients who experienced complete response (CR) or partial response (PR) were eligible for larynx preservation protocol. The corresponding patients were administrated for another PF/TP/TPF cycle, followed by intensity-modulated radiotherapy (one 2-Gy fraction per day,5 days per week, for a total of 70 Gy). Patients with stable disease (SD) or progressive disease (PD) underwent immediate salvage surgery. After larynx preservation treatment, voice quality and swallowing ability were assess using Functional Assessment of Cancer Therapy–Head and Neck Scale (10).

Acute toxicity was graded according to the National Cancer Institute-Common Toxicity Criteria for Adverse Events version 3.0. Late toxicity was graded according to the Radiation Therapy Oncology Group (RTOG) Late Radiation Morbidity Scoring Criteria.

### Observation End Points

Overall survival (OS) was calculated from the first day of treatment to the last follow-up or the date when the patient died from any cause. Progression free survival (PFS) was defined from the first day of treatment to the day of discovery of any tumor (local, regional, metastatic, or second primary) after treatment or death from any cause. Routine follow up were performed by personnel in the Clinical Follow-up Department of SYSUCC every six months and a final follow up was conducted by X. Su and C.Y. He. Patients were finally followed up until Aug 15, 2017. Patients were taken for censor if the defined event did not occur until the cutoff date.

### Statistical Analysis

The difference of distribution of category variable was compared by Chi-square test.

The 3-year, 5-year, and 10-year survival rates were calculated using a life table. The difference of PFS and OS were analyzed using the Kaplan–Meier method and tested by a log-rank test. The log-rank test was used for comparison in the Univariate analysis. The Cox proportional hazard regression model with an enter step was used for the multivariable analysis with the factors that reach significance in Univariate analysis. Their corresponding effects were evaluated in multivariable analysis. A P value < 0.05 was considered statistically significant. SPSS 16.0 software (SPSS Inc.; Chicago, IL, USA) was used for statistical analysis. The authenticity of this article has been validated by uploading the key raw data onto the Research Data Deposit public platform (www.researchdata.org.cn), with the approval RDD number as RDDA2020001507.

## Results

### Patient Characteristics

**Figure 1:**
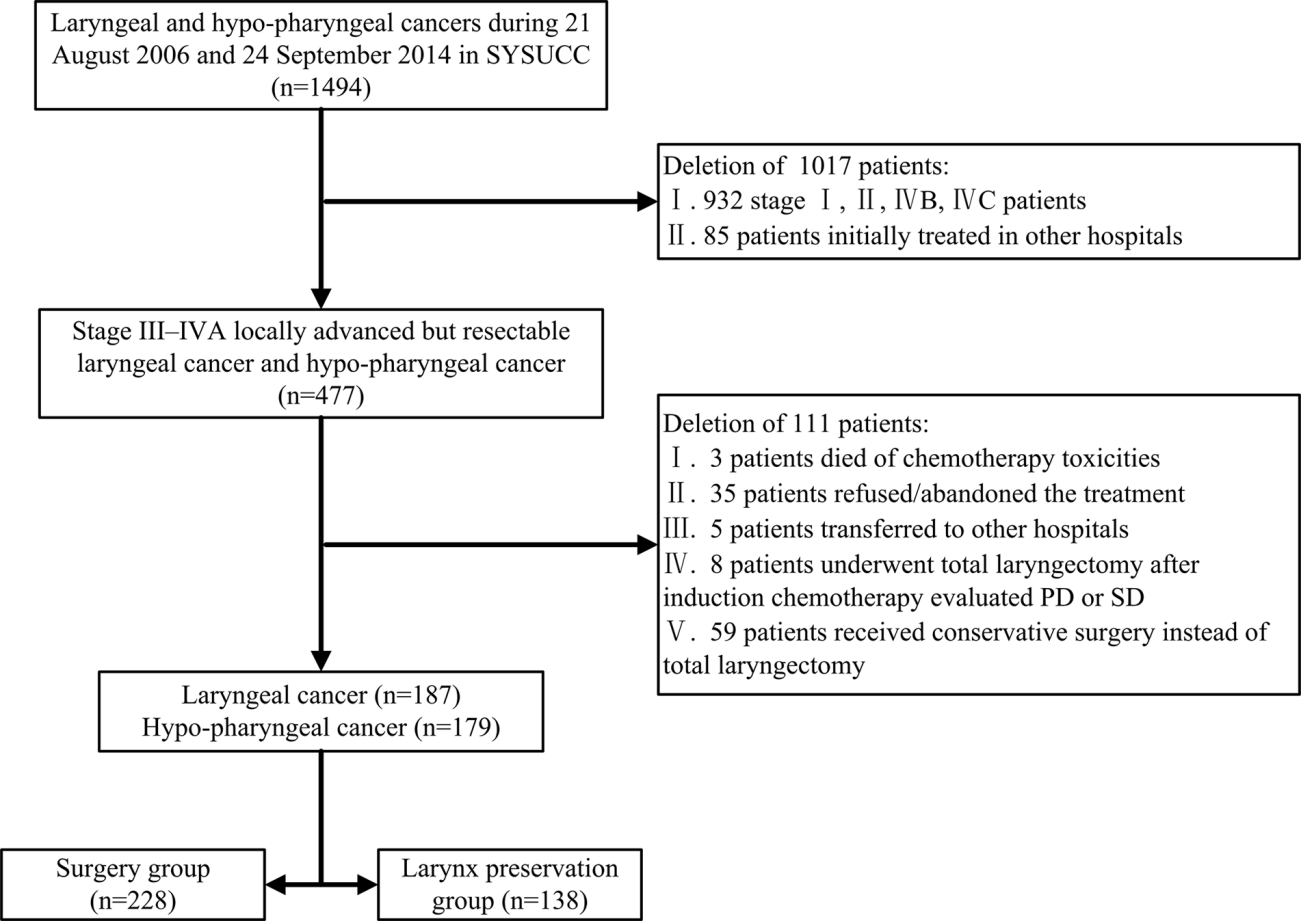
Screening flowchart for eligible patients.

Overall, the records of 1,494 patients with laryngeal cancer and hypopharyngeal cancer were reviewed, and 366 patients with locally advanced laryngeal cancer or hypopharyngeal cancer who received their initial treatment in SYSUCC were included in the analysis. The screening flowchart of eligible patients is shown in **Figure 1**. Patients’ mean age was 59.3 ± 9.7 years, ranging from 29 to 82 years. The majority of patients were male (97.0%). Among the them, 258 (70.5%) were smokers and 167 (45.6%) were alcohol drinkers. There were 187 patients with laryngeal cancer and 179 patients with hypopharyngeal cancer, 51 (13.9%) cases was in T2-stage, 135 (36.9%) in T3-stage and 180 (49.2%) in T4-stage. None of the patients had distant metastasis (all M = 0). The demographic and tumor characteristics of patients are shown on **Table 1**.

**Table 1:** Demographic and clinical characteristics of the patients with laryngeal and hypopharyngeal carcinoma.

Characteristics	All n =366	Surgery group n = 228	larynx preservation group n =138	P^a^
Sex				0.242
Male	355 (97.0%)	223 (97.8%)	132 (95.7%)	
Female	11 (3.0%)	5 (2.2%)	6 (43%)	
Age(years)				<0.010
Mean ± SD	59.3 ± 9.7	61.0 ± 9.3	56.5 ± 9.8	
Range	29-82	33-82	29-78	
Smoking				0.796
Yes	258 (70.5%)	161 (70.6%)	97 (70.3%)	
No	108 (29.5%)	67 (29.4%)	41 (29.7%)	
Alcohol consumption				0.264
Yes	167 (45.6%)	99 (43.4%)	68 (49.3%)	
No	199 (54.4%)	129 (56.6%)	70 (50.7%)	
Cancer types				<0.010
Laryngeal carcinoma	187 (51.1%)	141 (61.8%)	46 (33.3%)	
Hypopharyngeal carcinoma	179 (48.9%)	87 (38.2%)	92 (66.7%)	
Site for laryngeal carcinoma				0.129
Supraglottic region	49 (26.2)	33 (23.4%)	16 (34.8%)	
Glottic region	133 (71.1%)	105 (74.5%)	28 (60.9%)	
Subglottic region	5 (2.7%)	3 (2.1%)	2 (4.3%)	
Site for hypopharyngeal carcinoma				0.016
Pyriform sinus	164 (91.6%)	85 (97.7%)	79 (85.9%)	
Retropharyngeal wall	14 (7.8%)	2 (2.3%)	12 (13.0%)	
Postcricoid region	1 (0.6%)	0 (0.0%)	1 (1.1%)	
Stage^b^				0.010
III	161 (44.0%)	88 (38.6%)	73 (52.9%)	
IVa	204 (55.7%)	139 (61.0%)	65 (47.1%)	
T stage				0.001
T2	51 (13.9%)	21 (9.2%)	30 (21.7%)	
T3	135 (36.9%)	83(36.4%)	52 (37.7%)	
T4	180 (49.2%)	124(54.4%)	56 (40.6%)	
N stage				0.015
N0	119 (32.5%)	85(37.3%)	34 (24.6%)	
N1	69 (18.9%)	45 (19.7%)	24 (17.1%)	
N2	178 (48.6%)	98 (43.0%)	80 (58.0%)	

^a^The difference of distribution of category variable was compared by Chi-square test. ^b^one case was T2N0 subglottic laryngeal carcinoma, stage II but need total laryngectomy.

### Treatment

Total laryngectomy was performed in 228 patients, and 138 patients received larynx preservation treatments. Among the surgical group, 122 (53.5%) patients underwent total laryngectomy alone, 59 (25.9%) patients underwent total laryngectomy followed by radiotherapy (RT), and 47 (20.6%) patients underwent total laryngectomy followed by concomitant radio-chemotherapy (RT/CT). Among the patients receiving larynx preservation options, two (1.4%) patients received radical RT alone, 33 (23.9%) patients received RT/CT, and 103 (74.7%) patients received induction chemotherapy followed by RT (40 cases) or RT/CT (63 cases).

### Assessment of Larynx Function in Patients With Larynx Preservation Treatment

Those 138 patients with intent to preserve the larynx fulfilled the full course of larynx preservation treatment and were included in the subsequent analysis. The 1-year, 3-year, and 5-year larynx function preservation (LFP) were 89.2%, 85.0%, and 83.4%, respectively, when the cancer-death cases were excluded for the assessment of LFP. The 1-year, 3-year, and 5-year LFPs were 76.3%, 60.4%, and 54.0%, respectively, when the cases received a salvage surgery and cancer-death cases were considered as failed cases. The data on the assessment of voice quality and swallowing ability was not analyzed because the low number of questionnaires filled in.

### Prognostic Factors for Patients With Laryngeal Cancer and Hypopharyngeal Cancer

**Figure 2:**
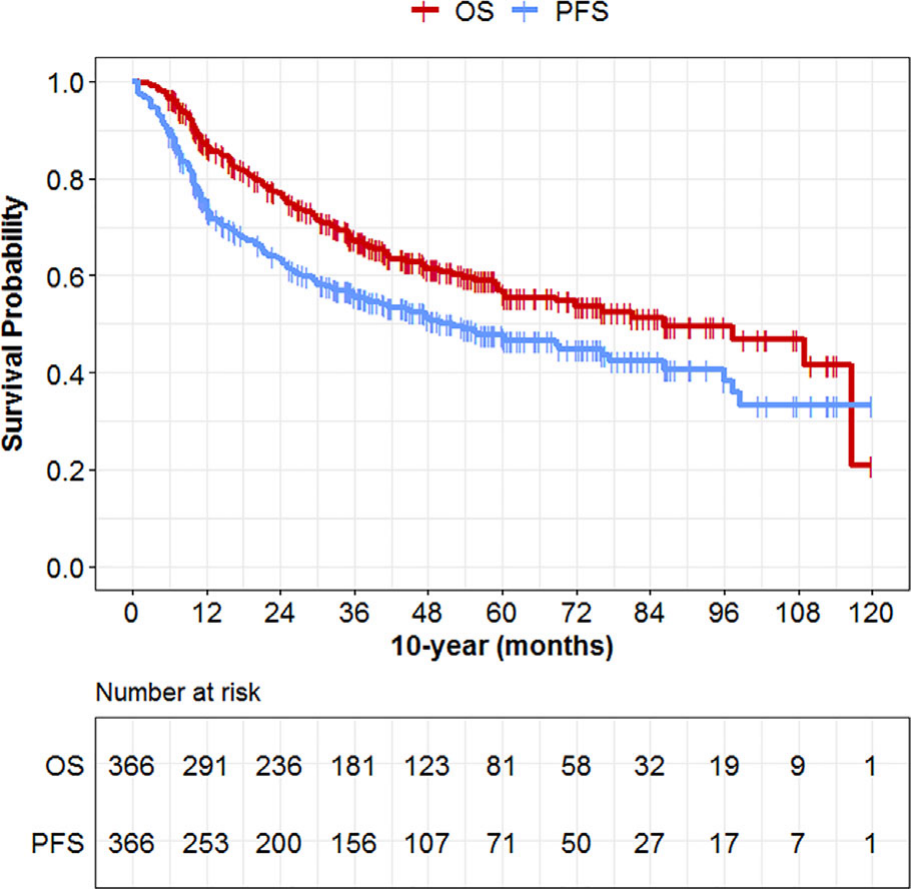
Overall survival data of the 366 patients with laryngeal and hypopharyngeal cancers.

With a median follow-up of 35.4 months (quartiles 25% and 75%: 15.0–57.2 months) for all patients, the overall 3-year, 5-year, and 10-year overall survival rates were 61%, 54%, and 31%, respectively (**Figure 2**). PFS rates were 51%, 44%, and 31%, respectively.

Among the clinical factors in the Univariate analysis, we found no significant differences of the OS and PFS in gender, age, smoking status, alcohol consumption, site of laryngeal or hypopharyngeal cancers, AJCC staging, T stage, and treatment group, as shown in **Tables 2** and **3**. In contrast, the cancer type and N stage had significant prognostic impacts for the OS and PFS in the overall patients (**Tables 2** and **3**). Efficacy assessment during induction chemotherapy had significant prognostic impacts for the OS and PFS in the patients receiving larynx preservation treatment (**Tables 2** and **3**).

**Table 2:** Univariate analysis of progression-free survival in patients with laryngeal and hypopharyngeal carcinoma.

Variable	3-year(%)	HR(95%CI)	P	5-year(%)	HR(95%CI)	P	10-year (%)	HR(95%CI)	P
Sex									
Female	51	1(ref)		/	1(ref)		/	1(ref)	
Male	51	1.11(0.41–3.00)	0.839	44	1.00(0.41–2.43)	0.993	31	1.07(0.44–2.60)	0.883
Age, years									
≤59	54	1(ref)		46	1(ref)		24	1(ref)	
>59	52	0.77(0.56–1.07)	0.117	43	0.82(0.61–1.12)	0.209	43	1.14(0.85–1.53)	0.386
Smoking									
No	53	1(ref)		44	1(ref)		44	1(ref)	
Yes	50	1.07(0.75–1.53)	0.723	44	1.09(0.77–1.54)	0.616	30	1.09(0.78–1.53)	0.626
Alcohol consumption									
No	51	1(ref)		44	1(ref)		26	1(ref)	
Yes	50	1.03(0.75–1.43)	0.844	43	1.04(0.77–1.42)	0.787	35	1.01(0.75–1.36)	0.965
Site for laryngeal cancer									
Subglottic region	75	1(ref)		75	1(ref)		/	1(ref)	
Glottic region	59	0.87(0.51–1.46)	0.591	52	0.79(0.49–3.58)	0.337	38	0.80(0.50–1.29)	0.361
Supraglottic region	50	0.59(0.08–4.36)	0.601	47	0.48(0.07–3.58)	0.476	16	0.48(0.07–3.52)	0.468
Site for hypopharyngeal cancer									
Pyriform sinus	46	1(ref)		38	1(ref)		32	1(ref)	
Retropharyngeal wall	33	1.67(0.83–3.34)	0.148	11	1.50(0.75–2.98)	0.252	/	1.67(0.86–3.22)	0.128
Postcricoid region	100	/	/	/			/	/	/
Stage									
III	52	1(ref)		42	1(ref)		26	1(ref)	
IV	50	1.17(0.85–1.62)	0.345	45	1.07(0.79–1.45)	0.662	35	1.04(0.77–1.40)	0.796
T stage									
T2	42	1(ref)		23	1(ref)		23	1(ref)	
T3	52	0.74(0.46–1.19)	0.212	47	0.71(0.45–1.10)	0.127	26	0.71(0.46–1.10)	0.121
T4	53	0.81(0.52–1.28)	0.367	47	0.73(0.48–1.12)	0.152	36	0.72(0.47–1.09)	0.123
N stage									
N0	63	1(ref)		58	1(ref)		40	1(ref)	
N1	56	1.79(1.07–2.99)	0.027	49	1.44(0.89–2.32)	0.140	32	1.43(0.90–2.27)	0.132
N2	41	2.74(1.82–4.15)	1.68×10^-6^	33	2.33(1.61–3.38)	7.99×10^-6^	27	2.30(1.61–3.30)	5.00×10^-6^
Treatment									
Surgery	54	1(ref)		48	1(ref)		33	1(ref)	
laryngeal preservation group	47	1.26(0.91–1.74)	0.167	36	1.22(0.90–1.67)	0.207	27	1.28(0.94–1.73)	0.115
Cancer type									
Laryngeal carcinoma	57	1(ref)		51	1(ref)		34	1(ref)	
Hypopharyngeal carcinoma	45	1.47(1.07–2.03)	0.019	35	1.50(1.11–2.04)	0.009	30	1.51(1.12–2.03)	0.006
Efficacy assessment of laryngeal preservation^a^									
No	37	1(ref)		/	1(ref)		/	1(ref)	
Yes	54	0.46(0.25–0.85)	0.012	50	0.54(0.30–0.97)	0.040	/	0.54(0.30–0.97)	0.040

a The effect of efficacy assessment during induction chemotherapy on the PFS was evaluated in the subpopulations who received the laryngeal preservation treatment. Cumulative survival rates were calculated with Kaplan-Meier method and differences between survival curves were calculated using the log-rank test. P < 0.05 were considered statistically significant. OS, overall survival; PFS, progression free survival.

**Table 3:** Univariate analysis of overall survival in patients with laryngeal and hypopharyngeal carcinoma.

Variable	3-year(%)	HR(95%CI)	P	5-year(%)	HR(95%CI)	P	10-year(%)	HR(95%CI)	P
Sex									0.526
Female	51	1(ref)		/	1(ref)		/	1(ref)	
Male	62	0.74(0.27–2.02)	0.562	54	0.71(0.29–1.74)	0.453	31	0.75(0.31–1.83)	0.528
Age, years									
≤59	61	1(ref)		54	1(ref)		54	1(ref)	
>59	62	0.78(0.54–1.15)	0.21	54	0.91(0.64–1.28)	0.580	18	0.98(0.70–1.37)	0.886
Smoking									
No	65	1(ref)		49	1(ref)		49	1(ref)	
Yes	61	0.88(0.58–1.34)	0.552	55	0.89(0.61–1.31)	0.550	28	1.10(0.76–1.61)	0.610
Alcohol consumption									
No	60	1(ref)		53	1(ref)		40	1(ref)	
Yes	63	1.21(0.82–1.78)	0.329	53	1.09(0.76–1.54)	0.652	25	1.07(0.76–1.50)	0.702
Site for laryngeal cancer									0.807
Subglottic region	100	1(ref)		100	1(ref)		/	1(ref)	
Glottic region	65	1.05(0.57–1.95)	0.879	57	0.90(0.53–1.53)	0.690	47	0.94(0.56–1.58)	0.817
Supraglottic region	60	/	/	54	0.52(0.07–3.85)	0.519	/	0.52(0.07–3.88)	0.525
Site for hypopharyngeal cancer									
Pyriform sinus	60	1(ref)		53	1(ref)		23	1(ref)	
Retropharyngeal wall	46	2.19(0.99–4.83)	0.053	23	1.89(0.86–4.150	0.112	23	2.03(0.97–4.25)	0.060
Postcricoid region	100			/			/	/	/
Stage									0.290
III	64	1(ref)		54	1(ref)		15	1(ref)	
IV	63	1.54(1.03–2.28)	0.034	53	1.29(0.91–1.85)	0.155	48	0.83(0.59–1.17)	0.291
T stage									
T2	63	1(ref)		41	1(ref)		41	1(ref)	
T3	60	0.93(0.51–1.70)	0.822	55	0.96(0.56–1.65)	0.874	9	1.01(0.61–1.68)	0.969
T4	61	1.15(0.65–2.04)	0.626	55	1.04(0.62–1.76)	0.885	49	0.97(0.68–1.40)	0.880
N stage									
N0	68	1(ref)		62	1(ref)		51	1(ref)	
N1	68	1.15(0.61–2.13)	0.664	56	1.11(0.64–1.92)	0.717	19	1.13(0.66–1.91)	0.662
N2	55	2.33(1.46–3.71)	3.88×10^-4^	47	1.85(1.23–2.78)	0.003	24	1.88(1.27–2.78)	0.002
Treatment									
Surgery	62	1(ref)		55	1(ref)		27	1(ref)	
laryngeal preservation group	60	1.12(0.76–1.66)	0.565	51	1.04(0.72–1.49)	0.836	40	1.09(0.77–1.55)	0.628
Cancer type									
Laryngeal carcinoma	63	1(ref)		56	1(ref)		36	1(ref)	
Hypopharyngeal carcinoma	59	1.32(0.90–1.94)	0.152	51	1.22(0.86–1.73)	0.257	22	1.29(0.92–1.81)	0.138
Efficacy assessment of laryngeal preservation^a^									
No	46	1(ref)		/	1(ref)		/	1(ref)	
Yes	69	0.34(0.17–0.68)	0.003	69	0.40(0.20–0.78)	0.008	/	0.40(0.20–0.78)	0.008

a The effect of efficacy assessment during induction chemotherapy on the OS was evaluated in the subpopulations who received the laryngeal preservation treatment. Cumulative survival rates were calculated with Kaplan-Meier method and differences between survival curves were calculated using the log-rank test. P < 0.05 were considered statistically significant. OS, overall survival; PFS, progression free survival.

A multivariable analysis (**Table 4**) was performed to determine which clinical or therapeutic variables were strongly correlated with OS and PFS. The cancer type and N stage that demonstrated a statistical significance in univariable analysis were included in the Cox proportional hazard regression model with an enter step. The N stage was found to be an independent prognostic indicator for PFS and OS in all the included patients. The N2-stage disease increased the risk of disease progression (HR = 2.16, 95%CI: 1.45–3.22, P = 1.51×10^-5^) and impaired the OS (HR = 1.86 95%CI: 1.20–2.90, P = 0.006).

**Table 4:** Multivariable analysis of prognostic factors for patient survival.

Variable	For PFS		For OS	
	HR(95%)	P value	HR(95%)	P value
Cancer type				
laryngeal carcinoma	1(ref)		1(ref)	
hypopharyngeal carcinoma	1.13(0.81–1.57)	0.469	1.01(0.69–1.48)	0.946
Lymph node metastasis				
N0	1(ref)		1(ref)	
N1	1.35(0.83–2.20)	0.230	1.12(0.64–1.94)	0.690
N2	2.16(1.45–3.22)	1.51×10^-5^	1.86(1.20–2.90)	0.006

Multivariable analysis was performed with the Cox proportional hazard regression model with an enter step in the total samples. The variables of cancer type and lymph node metastasis that demonstrated a statistical significance (P < 0.05) in univariate analysis were included and their corresponding effects were evaluated in multivariable analysis.

### Comparison of Progression Free Survival and Overall Survival Between the Surgery and Larynx Preservation Groups

**Figure 3:**
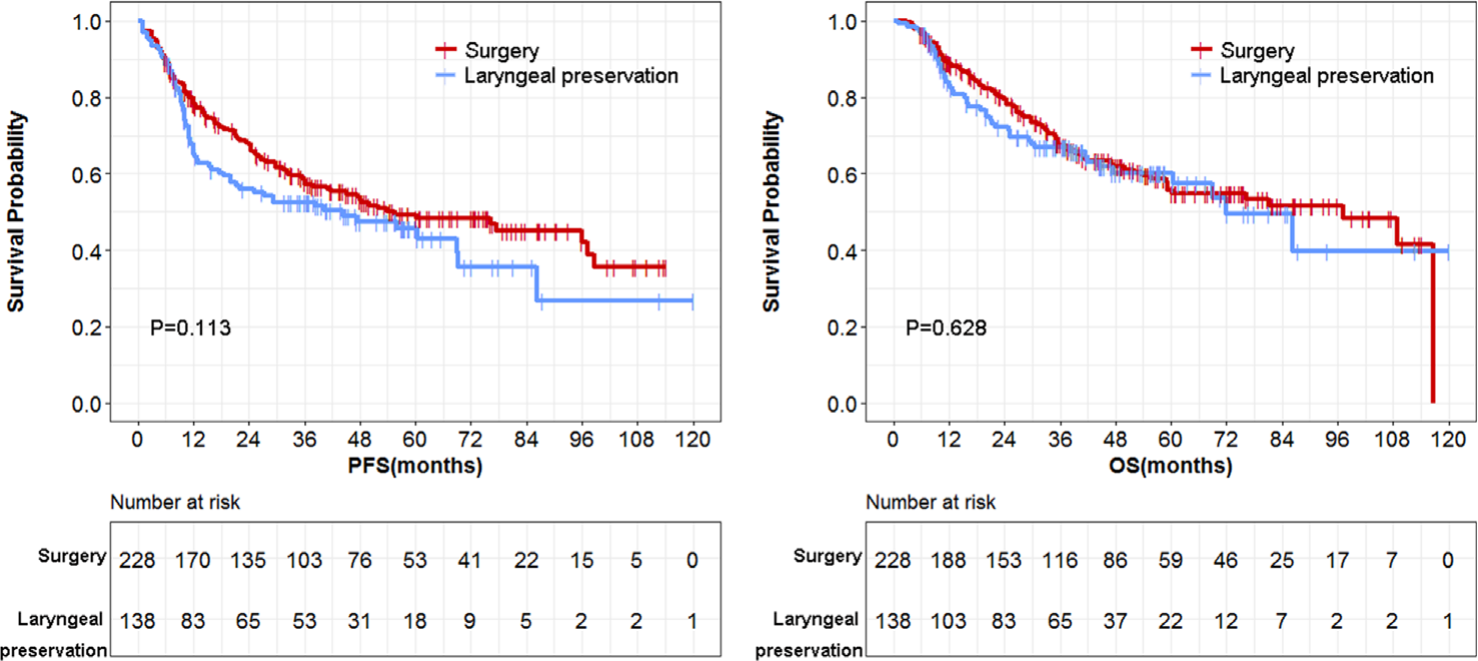
Comparison of overall survival data between surgery and larynx preservation groups.

Overall PFS and OS for the surgery and larynx preservation groups were analyzed in all subjects. There was no statistical difference in 3-year, 5-year, and 10-year PFS and OS in the comparison between the surgery and larynx preservation groups (**Table 5** and **Figure 3**).

**Table 5:** Univariate analysis of survival data between the surgery group and the larynx preservation group.

Variable	Surgery group vs. Laryngeal preservation group								
	3-year(%)	HR(95%CI)	P	5-year(%)	HR(95%CI)	P	10-year(%)	HR(95%CI)	P
**Analysis for OS**									
Total sample	62/60	0.89(0.60–1.32)	0.565	55/51	0.96(0.67–1.38)	0.836	27/40	0.92(0.65–1.30)	0.628
T stage									
T2	48/73	3.19(1.09–9.34)	0.035	31/52	3.06(1.15–8.19)	0.026	31/52	2.72(1.07–6.96)	0.036
T3	64/55	0.64(0.33–1.22)	0.176	59/41	0.71(0.39–1.27)	0.246	9/41	0.67(0.38–1.20)	0.177
T4	63/58	0.75(0.43–1.30)	0.301	58/53	0.85(0.50–1.44)	0.534	53/38	0.80(0.48–1.33)	0.379
N stage									
N0	69/65	0.95(0.39–2.28)	0.900	65/56	0.93(0.45–1.95)	0.855	55/33	0.77(0.39–1.54)	0.467
N1	70/63	0.69(0.25–1.95)	0.486	61/45	0.82(0.33–2.07)	0.679	20/45	0.71(0.29–1.72)	0.449
N2	52/58	1.12(0.69–1.82)	0.656	43/50	1.19(0.74–1.90)	0.468	12/50	1.21(0.77–1.91)	0.413
Cancer type									
Laryngeal carcinoma	64/63	0.86(0.46–1.61)	0.632	56/57	0.99(0.55–1.76)	0.959	30/41	0.93(0.54–1.61)	0.795
Hypopharyngeal carcinoma	59/59	1.04(0.62–1.76)	0.880	54/45	1.05(0.64–1.72)	0.842	22/45	1.00(0.62–1.61)	0.987
Site for laryngeal cancer									
Supraglottic region	58/64	1.13(0.36–3.62)	0.833	51/64	1.30(0.47–3.58)	0.614	0/64	1.30(0.47–3.58)	0.614
Glottic region	66/61	0.69(0.32–1.46)	0.328	59/50	0.77(0.38–1.56)	0.467	50/34	0.68(0.35–1.31)	0.245
Site for hypopharyngeal cancer									
Pyriform sinus	59/60	1.15(0.65–2.02)	0.631	54/51	1.13(0.67–1.92)	0.649	22/51	1.09(0.65–1.83)	0.740
Retropharyngeal wall	50/44	1.09(0.13–9.10)	0.940	/	/	/	/	/	/
**Analysis for PFS**									
Total sample	54/47	0.80(0.57–1.10)	0.167	48/36	0.82(0.60–1.12)	0.207	33/27	0.78(0.58–1.06)	0.115
T stage									
T2	34/48	1.32(0.60–2.89)	0.491	17/29	1.49(0.72–3.09)	0.284	17/29	1.41(0.69–2.88)	0.351
T3	60/40	0.47(0.27–0.81)	0.007	55/24	0.50(0.30–0.84)	0.009	31/24	0.48(0.29–0.80)	0.005
T4	53/55	1.05(0.63–1.75)	0.848	48/46	1.04(0.64–1.68)	0.887	37/31	0.99(0.62–1.58)	0.961
N stage									
N0	65/59	0.91(0.42–1.99)	0.817	61/49	0.87(0.44–1.72)	0.692	44/24	0.75(0.40–1.43)	0.385
N1	57/55	0.85(0.39–1.83)	0.845	53/37	0.89(0.41–1.92)	0.769	35/37	0.80(0.38–1.67)	0.545
N2	42/40	0.90(0.60–1.36)	0.615	33/31	0.93(0.63–1.39)	0.736	26/31	0.94(0.64–1.39)	0.761
Cancer type									
Laryngeal carcinoma	56/60	0.99(0.56–1.73)	0.968	51/53	1.06(0.62–1.81)	0.843	33/35	0.99(0.59–1.65)	0.967
Hypopharyngeal carcinoma	50/40	0.81(0.53–1.26)	0.355	42/26	0.83(0.55–1.25)	0.371	34/26	0.80(0.53–1.20)	0.271
Site for laryngeal carcinoma									
Supraglottic region	49/52	1.02(0.39–2.66)	0.966	45/52	1.01(0.50–2.01)	0.980	45/52	1.01(0.42–2.44)	0.980
Glottic region	58/62	0.89(0.44–1.79)	0.745	52/52	1.00(0.50–2.01)	0.993	37/34	0.90(0.48–1.71)	0.747
Site for hypopharyngeal carcinoma									
Pyriform sinus	50/41	0.86(0.54–1.36)	0.509	42/30	0.86(0.56–1.32)	0.488	34/30	0.84(0.55–1.29)	0.431
Retropharyngeal wall	50/100	0.79(0.10–6.33)	0.821	/	/	/	/	/	/

Cumulative survival rates were calculated with Kaplan-Meier method and differences between survival curves were calculated using the log-rank test. P < 0.05 were considered statistically significant. OS, overall survival; PFS, progression free survival.

**Figure 4:**
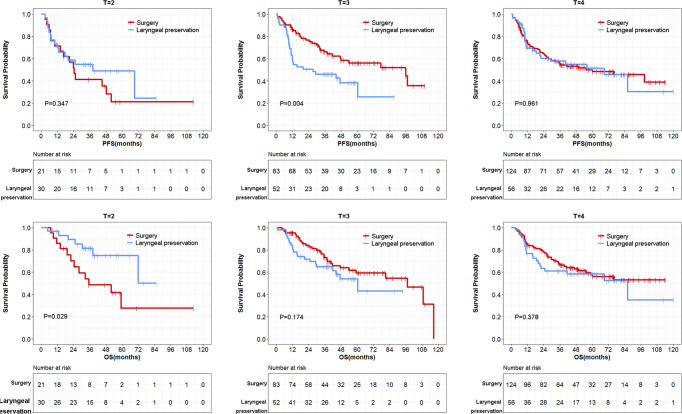
Stratification analysis of survival data by T stage between surgery and larynx preservation groups.

Further subgroup analysis by T stage, N stage, cancer type, and site of cancer was performed (**Table 5**). In the stratification analysis by cancer type, regardless of laryngeal cancer or hypopharyngeal cancer subpopulations, there was no difference of 3-year and 5-year PFS and OS were observed. These results indicated equally effective tumor control and survival between these two groups. Stratification analysis by N stage and site of cancers also showed negative results. In the stratification analysis by T stage (**Table 5** and **Figure 4**), similar between-group PFS and OS were observed for T4-stage disease. In T2-stage diseases, the larynx preservation group had a longer OS compared with the surgery group (**Table 5**). In contrast, in T3-stage disease, the surgery group showed a longer PFS compared with the larynx preservation group (**Table 5**). Nevertheless, there was no statistical difference of OS in these two groups. In addition, negative results concerning PFS and OS were found in the T4-stage subgroup (**Table 5**).

We further performed multivariable model including the factors of lymph nodal stage, cancer type and treatment. Neither the larynx preservation nor the surgery altered the OS in different T-stage subpopulations. However, it was observed that the treatment of surgery group benefited in PFS in T3-stage subpopulations (HR = 0.53, 95%CI: 0.31–0.91, P = 0.022) in the multivariable analysis.

### The Effect of Induction Chemotherapy in Larynx Preservation Treatment

Among the 138 patients who received larynx preservation treatment, the ICT/RT group did not exhibit better PFS and OS compared with the concurrent CT/RT group (**Table 6**). Similar results were found when separate analysis was performed for laryngeal cancer and hypopharyngeal cancer. We further explored the confounding factors for larynx preservation treatment.

**Table 6:** Analysis of survival data between larynx preservation treatments with or without induction chemotherapy.

Event	ICR/RT vs. CR/RT					
	3-year (%)	P	5-year (%)	P	10-year (%)	P
PFS	48/44	0.609	45/20	0.877	45/20	0.486
OS	62/56	0.900	61/35	0.909	61/35	0.520

Cumulative survival rates were calculated with Kaplan-Meier method and differences between survival curves were calculated using the log-rank test. P < 0.05 were considered statistically significant. ICR, induction chemotherapy; RT, radiotherapy; CR, chemotherapy; OS, overall survival; PFS, progression free survival.

**Figure 5:**
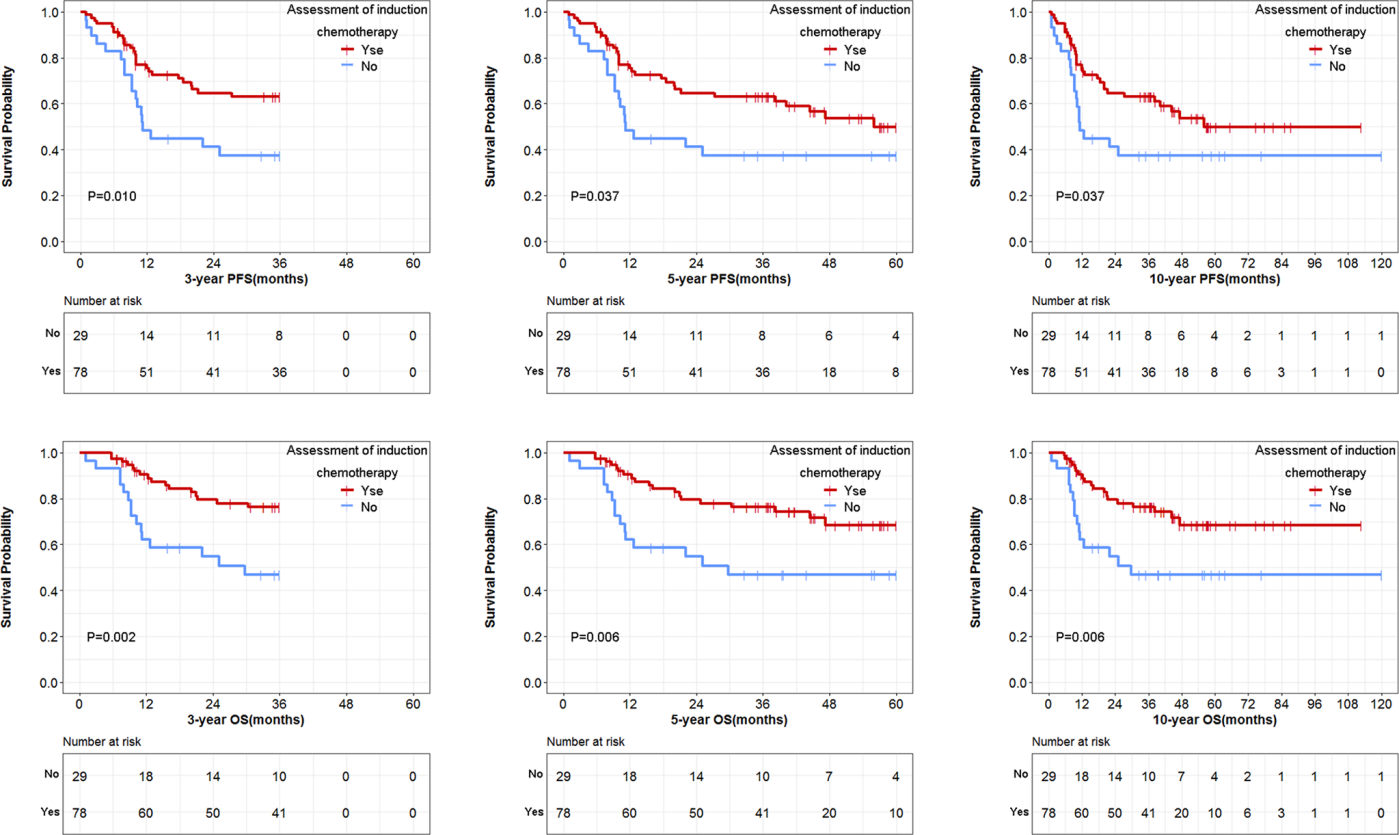
Comparison of survival data in induction chemotherapy treated groups, with or without efficacy assessment.

There were 103 patients who received ICT followed by RT or RT/CT. Among them, 76 underwent an efficacy assessment during the ICT treatment, while 27 cases did not undergo the same efficacy assessment. These patients were classified into two subgroups according to whether efficacy assessment was performed or not. The ICT subgroup with efficacy assessment demonstrated significantly longer 3-year, 5-year, and 10-year PFS than that of ICT subgroup without efficacy assessment (*P* = 0.010, 0.037 and 0.037, respectively; **Figure 5**). Consistently, longer 3-year, 5-year, and 10-year OS were observed in ICT subgroup with efficacy assessment (*P* = 0.002, 0.006 and 0.006; **Figure 5**) compared with the group without efficacy assessment.

**Figure 6:**
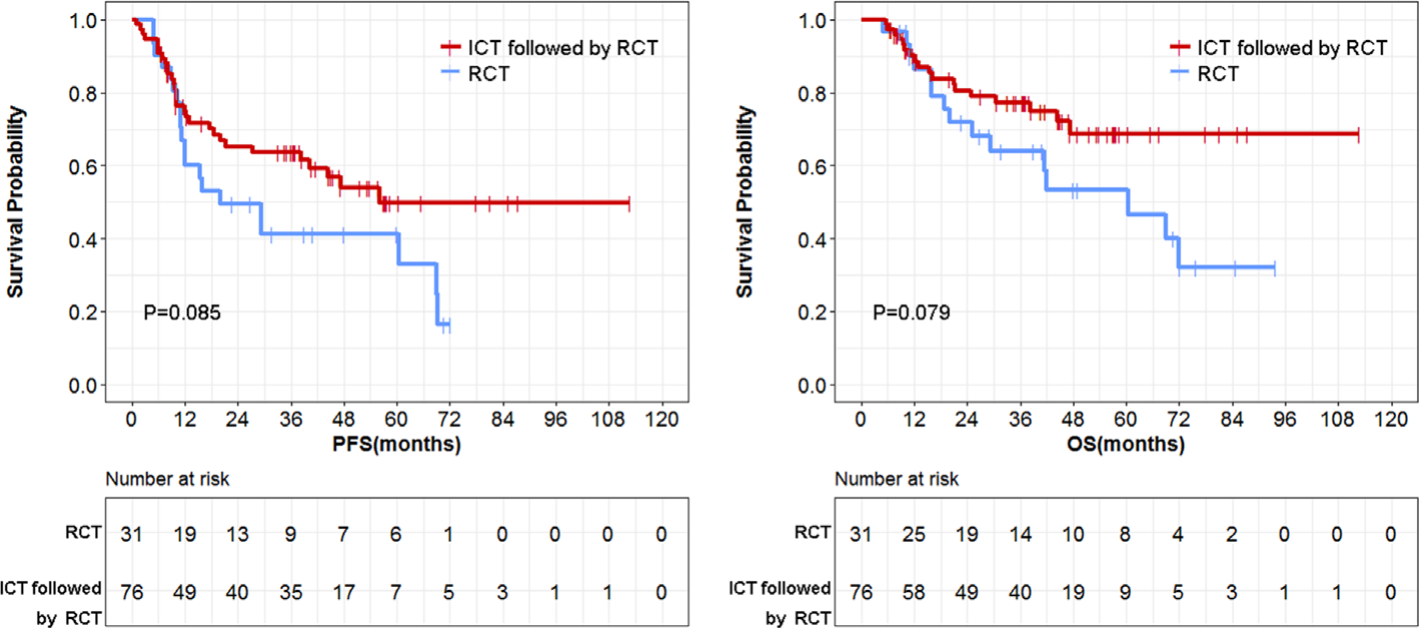
Comparison of survival data between induction chemotherapy/radiotherapy (CT/RT) group and concurrent CT/RT group.

Since efficacy assessment was found to be a confounding factor closely related to the prognosis of patients, we deleted the 27 cases that did not undergo an efficacy assessment during the ICT treatment and assessed its effect on the results of this study. The ICT/RT group did not show better PFS and OS compared with the concurrent CT/RT group (**Figure 6**) (*P* = 0.085 and 0.079, respectively). However, the difference of overall PFS and OS between the surgery and larynx preservation groups did not reach statistical significance (for 10-year PFS: HR = 1.17, 95%CI: 0.84-1.64, *P* = 0.345; for 10-year OS: HR = 0.96, 95%CI: 0.64–1.42, *P* = 0.819).

### Different Options in Induction Chemotherapy

**Figure 7:**
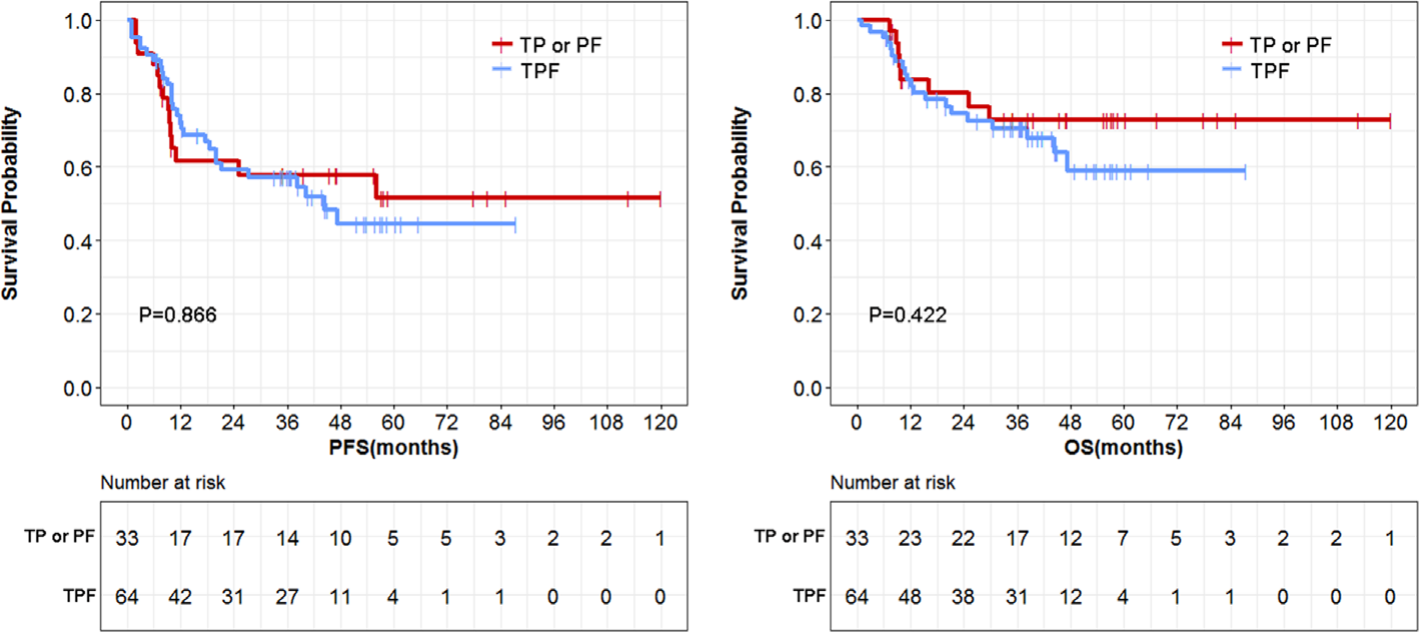
Comparison of survival data between triplet TPF group and doublet TP or PF group. PF, platinum plus fluorouracil; TP, docetaxel plus platinum; TPF, docetaxel, platinum and fluorouracil.

Among the 103 patients receiving full-course ICT, 14 patients received the PF regimen (platinum plus fluorouracil), 19 patients received the TP regimen (docetaxel plus platinum), 64 patients received the TPF regimen (docetaxel, platinum and fluorouracil), and 6 patients received other regimens. Data for patients receiving PF or TP regimens were combined because of the limited study samples. Patients receiving TPF regimens obtained similar PFS and OS compared with those receiving PF or TP regimens (**Figure 7**). TPF did not cause serious toxicities compared with FP and TP (**Table 7**).

**Table 7:** Toxicities induced by chemotherapy in patients receiving larynx preservation regimens.

Toxicities	PF/TP regimens					TPF regimens					P value
	Freq (%)	Degree of toxicity				Freq (%)	Degree of toxicity				
		I	II	III	IV		I	II	III	IV	
Vomiting	15 (45.5%)	10	5	0	0	34 (53.1%)	19	13	2	0	0.354
Stomatitis	7 (21.2%)	4	3	0	0	18 (28.1%)	9	6	3	0	0.450
Rash	2 (6.1%)	2	0	0	0	5 (7.8%)	5	0	0	0	/
Neutropenia	13 (39.4%)	8	2	2	1	30 (46.9%)	16	9	4	1	0.931
Thrombocytopenia	8 (24.2%)	3	5	0	0	17 (26.6%)	11	5	1	0	0.829
Anemia	9 (27.3%)	7	2	0	0	25 (39.1%)	17	8	0	0	1.000
Hypohepatia	3 (9.1%)	2	1	0	0	12 (18.8%)	10	2	0	0	0.516
Renal insufficiency	2 (6.1%)	2	0	0	0	3 (4.7%)	2	1	0	0	0.467

PF, platinum plus fluorouracil; TP, docetaxel plus platinum; TPF, docetaxel, platinum, and fluorouracil.

## Discussion

The 5-year OS of locally advanced laryngeal cancer declined over the past two decades, which might be correlated with the increased use of nonsurgical treatment (11). According to Cancer Statistics (2014), the 5-year OS for laryngeal cancer from 1975–1989 was 66%, while it was only 63% from 2002–2008 (12). However, it might be too easy to ascribe this declination to the application of non-surgical approaches. The observed decrease in survival in laryngeal cancer might have been partly caused by the use of radiation alone or the inappropriate administration of larynx preservation strategies in the early years (11).

The first guideline for treatment of laryngeal cancer with the intent of preserving the larynx was documented in 2006 (7). Since then, nonsurgical treatment strategies to preserve the larynx started to be introduced into China. To the best of our knowledge, this is the first study to report the effect of larynx preservation strategies in China. Regarding the therapeutic strategy (total laryngectomy vs. larynx preservation), no compromise in OS and PFS was observed in patients receiving the larynx preservation therapy. Consistently, Kim et al.’s study (13) compared the treatment results of locally advanced hypopharyngeal carcinoma according to treatment modalities, and found that nonsurgical therapy (ICT plus RT) was an effective strategy to achieve organ preservation without compromising survival. Moreover, we found that T2-stage laryngeal and hypopharyngeal cancers receiving larynx preservation treatment exhibited longer OS compared with surgery. The EORTC trials reported that patients with cancer of hypopharynx (T2 stage) were more likely to obtain complete response than T3 and T4 diseases in larynx preservation regimens (3), which partly support our findings. Pfister et al. recommended that all patients with T1 and T2 stage laryngeal cancer should be treated initially with intent to preserve the larynx (7). In contrast, our results showed that the option of surgery demonstrated better PFS in the T3-stage patients, although it had no contribution to overall survival. Overall, our data suggested that larynx preservation treatments did not jeopardize the survival of patients with laryngeal cancer or hypopharyngeal cancer. Due to the low number of subgroup analysis, whether T3-stage or T4-stage laryngeal and hypopharyngeal cancers patients are suitable for larynx preservation strategies needs further study.

There are many nonsurgical options available for organ and function preservation, that report discrepant effects in tumor control and survival (9, 11, 13). Although concurrent radiotherapy and chemotherapy has become the standard of care for larynx preservation (6, 14, 15), induction chemotherapy also demonstrates benefits in this disease (16). In 1987, a clinical study reported by Jacobs et al. pioneered the combination of induction chemotherapy and radiotherapy for advanced resectable laryngeal and hypopharyngeal cancers. This study suggested that patients with resectable disease who achieve a complete response to induction chemotherapy can be treated with primary radiation without compromising survival (17). The Department of Veterans Affairs Laryngeal Cancer Study Group first carried out a multicenter phase III randomized controlled clinical study on non-surgical treatment of laryngeal squamous cell carcinoma (2). Their results showed that induction chemotherapy followed by definitive radiation was an effective larynx preservation strategy. The EORTC phase III clinical study showed that compared with the surgery plus postoperative radiotherapy, induction chemotherapy followed by radiation can achieve similar outcome in patients with cancer of the hypopharynx while preserving laryngeal function (3, 4). But in RTOG 91-11 (Radiation Therapy Oncology Group) study, the authors did not find induction chemotherapy followed by radiotherapy superior to radiotherapy with concurrent administration of cisplatin, and they concluded that concurrent chemoradiotherapy should be considered as standard care for laryngeal cancer patients desiring laryngeal preservation. After follow-up of 10 years, induction PF followed by RT did not show better efficacy than concomitant cisplatin/RT for the composite end point of LFS. Concomitant cisplatin/RT has better locoregional control and larynx preservation than the induction arm or RT alone, but deaths that were not attributed to larynx cancer or treatment were higher (30.8% vs. 20.8% with induction chemotherapy and 16.9% with RT alone) (5). Whether induction chemotherapy follow by radiotherapy is better than concurrent chemoradiotherapy has not been reported. An ongoing French phase III trial (GORTEC 2014-03-SALTORL, clinicaltrials.gov NCT03340896) will provide an answer to this question.

In this study, we did not observe a better OS or PFS in induction chemotherapy followed by radiotherapy when compared with concurrent chemoradiotherapy. It is important to realize that only the standardized use of induction chemotherapy will benefit the patients. As observed in the present study, no efficacy assessment during the induction chemotherapy jeopardized the survival of patients. Since the early 1980s, cisplatin and 5-fluorouracil have been used in the patients with head and neck squamous cell cancers (16). Does the triplet therapy present better prognosis than the doublet therapy in the induction chemotherapy? The addition of TPF was documented to be more effective in prolonging survival than the doublet chemotherapy with cisplatin and 5-fluorouracil (18, 19). However, in the present study, we did not observe a statistical difference in PFS and OS between the TPF and PF or TP regimens. This deviation may have resulted from the small sample size of study subjects in the PF or TP groups in our study. The selection of appropriate induction therapy is very important to achieve optimal results for patients with intent to preserve the larynx (8). However, the sample sizes for these comparisons in this study were small and this issue deserves further investigation.

In this study, we combined laryngeal and hypopharyngeal cancers in the analysis when we evaluated the difference between surgery and larynx preservation treatment. Although a longer PFS and a similar OS in laryngeal cancer compared with hypopharyngeal cancer was observed, there was no statistical difference was found between surgery and larynx preservation treatment in the stratification analysis of cancer types. Concerning other studies, we found that although the prognosis of hypopharyngeal cancer is worse than laryngeal cancer, these two cancers have adjacent anatomical location, similarity in treatment strategies and effects on patients’ quality of life, these two types of cancers are often analyzed in combination. Actually, larynx preservation approach is applicable for both cancers in clinical practice.

There were several limitations in this study. First, we did not found the triple combination (TPF) to be more effective in prolonging survival than the doublet chemotherapy. This deviation may have resulted from the small sample size of study subjects in the PF or TP groups in our study. In addition, we found TPF regimen group (77%) has more patients with hypopharyngeal cancer compared with PF/TP regimens group (61%), which may also explain why TPF regimens was not found to be superior to the TP regimens in this study. Second, some patients did not received the efficacy assessment during the larynx preservation treatment. Our results highlighted an critical role of efficacy assessment for induction chemotherapy in achieving good outcomes for patients with intent to preserve the larynx. Third, this study was a retrospective, nonrandomized analysis of treatment results for patients with laryngeal and hypopharyngeal cancers. However, we comprehensively reviewed eligible cases that were treated at our institution over 10 years. In this study, we included patients from 2006 until 2014.

In conclusion, we confirmed that nonsurgical and surgical options are available in China for stage III-IVA resectable laryngeal and hypopharyngeal cancers. We noted that in earlier years, efficacy assessment during induction chemotherapy was neglected by certain physicians when patients were treated with induction chemotherapy, which reduce the benefits to patients. When performing induction chemotherapy, it should be kept in mind that efficacy assessment is essential to achieve the goal of larynx preservation without compromising ultimate tumor control and survival. We believe that this study will provide useful information for oncologists who make decisions on treatment options and modalities.

## Data Availability Statement

The raw data supporting the conclusions of this article will be made available by the authors, without undue reservation.

## Ethics Statement

The studies involving human participants were reviewed and approved by ethics committee of Sun Yat-Sen University Cancer Center. The patients/participants provided their written informed consent to participate in this study.

## Author Contributions

Conception and design: A-KY, C-YH. Development of methodology: XS, H-CH, Z-LY. Acquisition of data: QL, D-LZ, X-HY, Y-KL, TT, J-JM, B-HX, W-CC. Analysis and interpretation of data: XS, H-CH. Writing, review, and/or revision of the manuscript: XS, H-CH, Z-LY. Administrative, technical, or material support: XS, H-CH, Z-LY. Study supervision: A-KY, C-YH. All authors contributed to the article and approved the submitted version.

## Funding

This work was supported by the National Natural Science Foundation of China [grant number 81602426], the Natural Science Foundation of Guangdong Province [grant number 2017A030313865 and 2016A030310198], the Science and Technology Planning Project of Guangdong Province [grant number 2016A020215082 and 2014A020212100], and the Medical Science and Technology Research Fund Project of Guangdong Province [grant number A2015003].

## Conflict of Interest

The authors declare that the research was conducted in the absence of any commercial or financial relationships that could be construed as a potential conflict of interest.
